# Nicotine, Alcohol Consumption, and Risk of Myasthenia Gravis: Results From the Swedish Nationwide GEMG Study

**DOI:** 10.1212/WNL.0000000000213771

**Published:** 2025-06-10

**Authors:** Malin Petersson, Daniel Jons, Amalia Feresiadou, Andreea Ilinca, Fredrik Lundin, Rune Johansson, Anna Budzianowska, Anna-Karin Roos, Viktor Kagstrom, Martin Gunnarsson, Peter Sundström, Lars Klareskog, Tomas Olsson, Ingrid Kockum, Fredrik Piehl, Lars Alfredsson, Susanna Brauner

**Affiliations:** 1 Department of Clinical Neuroscience, Karolinska Institutet, Stockholm, Sweden;; 2 Department of Clinical Neuroscience, Institute of Neuroscience and Physiology, the Sahlgrenska Academy, University of Gothenburg, Sweden;; 3 Department of Neurology, Sahlgrenska University Hospital, Gothenburg, Sweden;; 4 Department of Neuroscience, Neurology, Uppsala University, Sweden;; 5 Department of Neurology, Skåne University Hospital, Malmö, Sweden;; 6 Division of Neurology, Department for Clinical Sciences, Lund University, Malmö, Sweden;; 7 Department of Neurology and Department of Biomedical and Clinical Sciences, Division of Neurobiology, Linköping University, Sweden;; 8 Department of Neurology and Rehabilitation, Karlstad Central Hospital, Sweden;; 9 Department of Neurology, Ryhov Hospital, Jönköping, Sweden;; 10 Unit of Neurology, Department of Clinical Science, Neurosciences, Umeå University, Östersund, Sweden;; 11 Rehabilitation Clinic, Sundsvall Hospital, Sweden;; 12 Department of Neurology, Faculty of Medicine and Health, Örebro University, Sweden;; 13 Department of Clinical Sciences, Neurosciences, Umeå University, Sweden;; 14 Department of Medicine, Karolinska Institutet, Stockholm, Sweden;; 15 Department of Neurology, Karolinska University Hospital, Stockholm, Sweden; and; 16 Institute for Environmental Medicine, Karolinska Institute, Stockholm, Sweden.

## Abstract

**Background and Objectives:**

Myasthenia gravis (MG), an autoimmune disease characterized by fluctuating muscle weakness, is believed to result from complex gene-environment interactions, yet few risk factors have been identified. The objective of this study was to determine the effect of nicotine and alcohol on MG disease risk.

**Methods:**

The Genes and Environment in Myasthenia Gravis study is a Swedish, nationwide cross-sectional case-control study where prevalent patients with MG were invited to submit an extensive questionnaire on lifestyle and environment. Data collection took place between November 2018 and August 2019, and cases were matched by sex and year of birth to population controls. Year of disease onset was used as index year. Associations between use of alcohol, tobacco smoke, Swedish snuff, and MG risk were investigated using multivariable logistic regression.

**Results:**

A total of 1,067 patients with MG (mean age at onset 48 (SD 21) years, 53% female) were matched to 2,087 controls. Any alcohol consumption was associated with a lower MG risk compared with not drinking at all (odds ratio [OR] 0.48, 95% CI 0.39–0.59, *p* < 0.001, exposed cases n = 616). Effects were observed in a similar direction across disease subtypes, with the strongest association in the late-onset MG group (onset ≥50 years). Although neither cigarette smoke nor use of Swedish snuff affected the disease risk of the whole group, subset specific effects were observed. Smoking at onset was associated with an increased risk of early-onset MG (EOMG, onset 18–49 years; OR 1.60, 95% CI 1.17–2.20, *p* = 0.003, n = 133), which was accentuated in acetylcholine receptor antibody–positive EOMG (OR 2.08, 95% CI 1.34–3.25, *p* = 0.001, n = 74). Use of Swedish snuff, which contains high levels of nicotine, at disease onset was also associated with an increased risk of EOMG (OR 1.61, 95% CI 1.02–2.54, *p* = 0.039, n = 43).

**Discussion:**

We observed an inverse correlation of MG risk and alcohol consumption. Furthermore, smoking and the use of Swedish snuff at disease onset were positively associated with EOMG. We recognize limitations related to retrospective data and limited number of available controls. However, multiple sensitivity analyses were performed supporting the robustness of our results.

## Introduction

Myasthenia gravis (MG) is a rare autoimmune neuromuscular disorder characterized by a variable degree of fluctuating muscle weakness.^1^ Symptoms are caused by autoantibodies directed toward receptors of the postsynaptic endplate, most often the nicotinergic acetylcholine receptor (AChR+), which are found in 80% of patients with MG.^1^ The most common MG subforms are early-onset MG (EOMG) and late-onset MG (LOMG), together accounting for approximately 70% of MG cases.^1^ EOMG usually starts in the second to fourth decades of life, predominantly affects women, and is associated with thymic hyperplasia and with an increased risk of developing other autoimmune diseases.^2^ LOMG affects men slightly more often than women, usually starts around 70 years of age, and is not associated with thymus pathology.^2^

The incidence and prevalence of MG has increased substantially during the past 2 decades, especially among older individuals.^3-5^ The cause is probably multifactorial with a combination of improved diagnostics, increased awareness, and longer life expectancy. However, changes in environmental and lifestyle risk factors may also contribute.^3-6^ MG is considered a complex disease, where both genetics and environmental factors have been associated with disease susceptibility.^2^ The genetic component is well established, primarily mediated by a strong HLA association. However, the genetic associations differ between EOMG and LOMG, where EOMG primarily is associated with the HLA-B*08:01 locus and LOMG with HLA-DRB1*15:01.^7^ Furthermore, changes in the genetic locus of *CTLA4*, a gene encoding an immune checkpoint receptor expressed on T cells, have been associated with EOMG.^8^ In LOMG, there are associations with *HLA-DQA1*, as well as *CHRNA1* (encoding the alpha subunit of the acetylcholine receptor) and *TNFRSF11A* (encoding RANK).^8,9^ Both heritability and monozygotic concordance rates have been estimated to around 35%, implicating a major effect on disease risk from environmental factors and gene-environment interactions.^8,10^ At present, the postpartum period is the only factor that has been associated with increased risk of disease development in patients with EOMG,^11^ while other factors remain unidentified.

Cigarette smoke is a well-established risk factor for both disease development and disease severity in autoimmune diseases such as multiple sclerosis (MS), rheumatoid arthritis (RA), and systemic lupus erythematosus (SLE).^12-16^ Although the exact mechanism by which smoking increases disease risk remains to be established, chronic inflammation of the airways due to the exposure to toxic compounds has been suggested to contribute.^17^ Swedish snuff is a ground oral tobacco, which contains more or at least as much nicotine per dose as a cigarette.^18^ Unlike smoking, oral snuff has been inversely associated with MS risk, whereas no clear effects have been observed in RA.^19-21^ To what degree smoking and nicotine use affects MG disease risk is not known. However, a higher proportion of smokers was found in both a prevalent Norwegian study and a smaller prevalent Swedish LOMG study, when comparing prevalent cases with population-based controls.^22,23^ By contrast, alcohol consumption has been inversely associated with both MS and RA.^24-27^ At present, there are however no studies addressing the role of alcohol in MG.

The Genes and Environment in Myasthenia Gravis (GEMG) case-control study was conducted to investigate the role of environmental and lifestyle factors in relation to MG disease risk and further disease development. The objective of this study was to explore potential associations between alcohol consumption, cigarette smoke, and Swedish snuff on the risk of developing MG.

## Methods

### Study Population

The GEMG study is a nation-wide study comprising 1,077 patients with MG,^28^ corresponding to about 42% of the prevalent MG population in Sweden.^3^ In short, patients with MG aged 18 years or older were identified through 3 means: (1) the national Swedish MG registry, (2) individuals with ≥2 inpatient or outpatient visits with the ICD code G70.0 recoded within the previous 10 years at the 12 largest neurology units in Sweden, and (3) by advertising through the Swedish MG patient organization. Identified individuals were subsequently invited from November 2018 until August 2019 to participate by answering an extensive questionnaire containing 106 questions regarding lifestyle and environmental factors during their life up until inclusion (see eMethods for translation of alcohol and tobacco-related questions). Information on serology was collected by linking the patients to the Swedish MG registry, a physician-reported voluntary national registry. Participants with insufficient Swedish reading proficiency were excluded, as were those with self-reported symptom onset before age 13, to minimize the risk of including congenital myasthenic syndromes.

Controls were selected from a pool of participants recruited for previous similar population-based studies conducted at Karolinska Institutet from 1994 and onwards investigating risk factors for MS (Epidemiological Investigation of Multiple Sclerosis [EIMS], Gene and Environment in Multiple Sclerosis [GEMS], and Immunomodulation and Multiple Sclerosis Epidemiology [IMSE]) and RA (Epidemiological Investigation of Rheumatoid Arthritis [EIRA]).^29-31^ The questions used in the GEMG study are very similar to the questions in GEMS and IMSE questionnaires with only minor differences to the EIMS questionnaire. EIRA only contains information on alcohol consumption in relation to inclusion, and controls from EIRA were therefore excluded in analyses containing alcohol as a covariate and only used in the case of unsuccessful matching to controls from the other studies, primarily for LOMG in the oldest age groups. Questions regarding Swedish snuff use and cigarette smoke were identical across all questionnaires.

Each MG case was matched to controls based on sex and year of birth ±2 years. We did not, in the primary analysis, match by year of case onset to year of control inclusion due to the large spread of onset and lack of controls included before the 1990s. Instead, controls had to be at least the same age at inclusion (in their study, respectively) as the matched case was at onset (index age). We aimed to match 2 controls to each case. If multiple controls met the matching criteria, priority was given to the studies that had the questionnaire most similar to GEMG. If several controls met the matching criteria within the same study, the selected controls were chosen using a pseudorandom generator. To assess the potential risk of survival bias, we investigated follow-up data, which were available for the MS controls (87% of all controls) at the time of study recruitment. Among the controls included in our study, >95% were alive at study recruitment (99% of the controls for EOMG and 91% for LOMG cases), indicating low probability of survival bias (data not shown).

### Definition of Exposures

Year of MG onset was set as index year for cases and their corresponding controls. The onset year was based on self-reported data, which, for this data set, have been externally validated previously showing reliable results (mean Cohens κ 0.93).^28^ If year of onset was not reported, year of diagnosis was used as a proxy. Exposures before or during onset were analyzed. The year the participant started and quit (if applicable) smoking or using Swedish snuff, respectively, was reported, along with the average daily number of cigarettes or weekly number of boxes of snuff. Exposure to passive smoke was reported in year the exposure began and ended. Smoking or use of oral snuff during the index year was classified as current use, participants who had stopped before the index year were classified as prior users, and those who reported no use before index as never users. Exposure to passive smoking was classified into ever if the participant had been exposed before index and current if the participant was currently exposed during the index year.

Alcohol consumption was reported as standard glasses per week during prespecified age intervals (15–19, 20–29, 30–39, 40–49, 50–59, and ≥60 years). Alcohol habits were assessed in the age category before the onset age to limit the influence of change of habits due to potential prodromal symptoms preceding symptom onset. Participants who, during the period before onset, had reported any alcohol consumption were defined as drinkers and participants who reported no alcohol use as nondrinkers. Furthermore, based on the amount of alcohol intake per week, as defined by Statistics Sweden, participants were classified as having low (<50 g/wk for women and <100 g/wk for men), moderate (50–112 g/wk for women and 100–168 g/wk for men), and high consumption (>112 g/wk for women and >168 g/wk for men).^32^ A standard glass was assumed to contain 12 grams of alcohol.^33^

Patients were divided into subgroups: EOMG (i.e., reported onset <50 years of age), LOMG (i.e., reported onset ≥50 years of age), and thymoma-associated MG (TAMG). The TAMG group (n = 45) was included in the overall analyses but was considered too small to be included in separate subgroup analyses. When defining EOMG and LOMG groups, we did not take antibody status into account but instead included AChR status (positive/negative) as an additional subgroup analysis within EOMG and LOMG separately as well as within the entire MG population.

### Statistical Methods

Conditional logistic regression was used to calculate odds ratios (ORs) with 95% CI. The dependent variable in the analyses was case-status (case/control), and the independent variables were smoking status, exposure to passive smoke, use of snuff, and alcohol use.

We undertook multiple sensitivity analyses, restricting the analysis to cases, and their corresponding controls, with onset within 5 years before inclusion (to decrease memory bias); AChR+ patients and stratified by subgroup (sensitivity analysis 1, eTables 1–3). In further sensitivity analyses, adjustments were made for geographical location at inclusion, adolescent body mass index, occupation, and university education, but as it only influenced the estimates minimally, it was not kept in the final analysis (eTable 4). We performed a sensitivity analysis by matching inclusion age and year of the controls to the cases' onset age and calendar year (±2 years), n cases = 793 (sensitivity analysis 2, eTables 1–3). In addition, due to the introduction of a smoking ban in restaurants in Sweden in 2005, we compared the rates off snuff users and the risk of using snuff before and after 2005 (eTable 5).

All analyses were performed using R version 4.1.3 and RStudio version 2023.12.1. Complete case analysis was performed, excluding cases and controls if variables were missing. Statistical significance was defined as *p* < 0.05. Owing to the exploratory nature of the study, no correction for multiple testing was performed.

### Standard Protocol Approvals, Registrations, and Patient Consents

The study was approved by the Regional Ethics Committee Stockholm, Sweden (2018/1436-31), and all participating patients gave written consent. The study was conducted in accordance with the Strengthening the Reporting of Observational Studies in Epidemiology guidelines.

### Data Availability

Deidentified data relating to the study are available from the corresponding author on reasonable request and ethical approval.

## Results

Of the 1,077 patients with MG included in the GEMG study, 1,067 were individually matched to a total of 2,087 controls, obtained from the GEMS (70%), IMSE (16%), and EIRA (13%) studies (eTable 6). Ten patients with MG remained unmatched due to missing data on disease onset and/or diagnosis (n = 4) or due to a combination of high onset age and early birth year (n = 6) and were therefore excluded. Women constituted 53% of the cases (n = 564). The mean age at onset was 48 years (SD 21) with 47% classified as EOMG, 48% LOMG, and 4.2% TAMG (Table 1). Time from index to study inclusion was longer in cases (mean 16 years, SD ±15) compared with controls (10 years; SD ±13). Almost two-thirds, 64%, of patients with MG reported regular alcohol consumption before onset, 42% were current or previous smokers, and 14.7% were ever-users of Swedish snuff. Serologic information was available for 70% of the cases of whom 84% (n = 628) were AChR+ (eTable 7).

**Table 1:** Basic Characteristics

Characteristic	Case (N = 1,067)	Control (N = 2,087)	EOMG (N = 505)	LOMG (N = 517)
Female	564 (53)	1,128 (54)	382 (76)	154 (30)
AChR				
POS	628 (84)		272 (79)	322 (86)
NEG	123 (16)		71 (21)	51 (14)
Unknown	316		162	144
Age at inclusion, y	64 (16)	58 (15)	54 (15)	74 (8)
Index age, y	48 (21)	47 (20)	29 (10)	66 (8)
Time from index to inclusion, y	16 (15)	10 (13)	25 (16)	8 (6)
Year of birth	1954 (16)	1954 (15)	1964 (15)	1944 (8)
University education at inclusion	238 (22)	444 (21)	128 (25)	98 (19)
Smoking status at onset				
Never	613 (58)	1,171 (58)	320 (64)	268 (52)
Current	170 (16)	318 (16)	133 (26)	30 (5.8)
Quit	278 (26)	531 (26)	50 (9.9)	215 (42)
Unknown	6	67	2	4
Snuff use at onset				
Never	906 (85)	1,737 (86)	451 (90)	419 (81)
Current	96 (9.0)	162 (8.0)	43 (8.5)	46 (8.9)
Quit	61 (5.7)	132 (6.5)	9 (1.8)	50 (9.7)
Unknown	4	56	2	2
Passive smoke exposure at onset				
Never	464 (44)	915 (45)	242 (49)	202 (40)
Current	121 (12)	268 (13)	99 (20)	19 (3.8)
Quit	462 (44)	846 (42)	157 (32)	285 (56)
Unknown	20	58	7	11
Alcohol use before onset				
None	48 (5.0)	114 (7.2)	169 (43)	157 (30)
Low	450 (47)	822 (52)	159 (40)	267 (52)
Moderate	118 (12)	261 (17)	44 (11)	71 (14)
High	340 (36)	384 (24)	23 (5.8)	22 (4.3)
Unknown	111	506	110	0

Abbreviations: EOMG = early-onset myasthenia gravis (onset 18–49 years); LOMG = late-onset myasthenia gravis (onset ≥50 years); TAMG = thymoma-associated myasthenia gravis.

Basic characteristics of cases and controls. Basic characteristics of cases, controls, and the subgroups EOMG, LOMG, and TAMG. Continuous values are presented as mean (SD) and categorical values as n (% of nonmissing values).

An inverse association between alcohol use (drinker vs nondrinker) and MG risk was observed in the whole group (OR 0.48, 95% CI 0.39–0.59, *p* < 0.001, exposed cases n = 616) (Table 2). Similar results were observed in both EOMG and LOMG, however, with a stronger effect in the LOMG group. We further observed a dose-dependent relationship most pronounced in LOMG, with a more than halved point estimate between low (OR 0.44, 95% CI 0.30–0.61, *p* < 0.001, n = 267) and high intake groups (OR 0.2, 95% CI 0.10–0.38, *p* < 0.001, n = 22) compared with nondrinkers. Finally, with each weekly unit alcohol increase, a significant risk reduction was observed (OR 0.97, 95% CI 0.95–0.99, *p* = 0.002). In sensitivity analyses either restricting to cases and controls with disease onset within 5 years before inclusion or when matching cases' onset year and age to controls' inclusion year and age, similar results were observed (eTable 1).

**Table 2:** Association Between Alcohol Use and MG Disease Development

	All			EOMG			LOMG		
	OR	95% CI	*p* Value	OR	95% CI	*p* Value	OR	95% CI	*p* Value
Drinker/nondrinker	0.48	0.39–0.59	<0.001	0.53	0.40–0.71	<0.001	0.41	0.30–0.56	<0.001
Alcohol consumption									
None	Ref 1.0			—			—		
Low	0.51	0.41–0.63	<0.001	0.57	0.41–0.77	<0.001	0.44	0.31–0.61	<0.001
Moderate	0.44	0.33–0.58	<0.001	0.43	0.28–0.66	<0.001	0.43	0.28–0.65	<0.001
High	0.35	0.23–0.53	<0.001	0.55	0.31–0.99	0.047	0.20	0.10–0.38	<0.001
Standard unit per week	0.97	0.95–0.99	0.002	1.00	0.98–1.01	0.61	0.95	0.92–0.97	<0.001

Abbreviations: EOMG = early-onset myasthenia gravis (onset 18–49 years); LOMG = late-onset myasthenia gravis (onset ≥50 years); MG = myasthenia gravis; OR = odds ratio.

Analyses were adjusted for smoking, exposure to passive smoke, and snuff use at onset using conditional logistic regression considering the matching variables sex, age at onset, and year of birth.

Ever smoking before onset was not associated with the occurrence of MG (OR 1.05, 95% CI 0.87–1.28, *p* = 0.60, n = 448), while smoking at onset showed an increased but nonsignificant risk (OR 1.26, 95% CI 0.98–1.61, *p* = 0.072, n = 170) in the whole cohort (Table 3). When restricting the analysis to AChR+ MG, a significant association was observed for smoking at onset (OR 1.60, 95% CI 1.13–2.26, *p* = 0.007, n = 94). In subgroup analysis, smoking at onset was associated with an increased occurrence of EOMG (OR 1.60, 95% CI 1.17–2.20, *p* = 0.003, n = 133), also here a stronger effect was observed in AChR+ EOMG (OR 2.08, 95% CI 1.34–3.25, *p* = 0.001, n = 74). By contrast, no association between smoking at onset and occurrence of LOMG was observed (OR 0.69, 95% CI 0.43–1.11, *p* = 0.13, n = 30), nor of AChR+ LOMG (OR 0.83, 95% CI 0.42–1.64, *p* = 0.59, n = 14). Similar observations, but less precise, were found in the sensitivity analyses, potentially due to smaller groups and reduced power (eTable 2).

**Table 3:** Association Between Cigarette Smoke and MG Disease Development

	All			EOMG			LOMG		
	OR	95% CI	*p* Value	OR	95% CI	*p* Value	OR	95% CI	*p* Value
Smoking at index	1.26	0.98–1.61	0.072	1.60	1.17–2.20	0.003	0.69	0.43–1.11	0.13
AChR+	1.60	1.13–2.26	0.007	2.08	1.34–3.25	0.001	0.83	0.42–1.64	0.59
Ever smoking	1.05	0.87–1.28	0.60	1.12	0.85–1.47	0.44	0.90	0.67–1.20	0.47
AChR+	1.18	0.91–1.54	0.21	1.28	0.87–1.89	0.21	1.01	0.68–1.49	>0.9

Abbreviations: EOMG = early-onset myasthenia gravis (onset 18–49 years); LOMG = late-onset myasthenia gravis (onset ≥50 years); MG = myasthenia gravis; OR = odds ratio.

All analyses are conditional logistic regression considering the matching variables sex, age at onset, and year of birth. Smoking at onset and ever smoking were adjusted for snuff use and passive smoke exposure at either onset or ever as well as alcohol consumption.

Swedish snuff contains high levels of nicotine but does not cause exposure to toxic inhaled compounds associated with airway irritation. Ever use of snuff was neither associated with MG overall (OR 1.14, 95% CI 0.87–1.49, *p* = 0.36, n = 157) nor in any of the subgroups or in sensitivity analyses (eTable 3). However, similar to smoking, use of snuff at onset was associated with an increased risk of EOMG (OR 1.61, 95% CI 1.02–2.54, *p* = 0.039, n = 43) (Table 4). An association between snuff use at onset and overall MG risk was evident also when the analysis was restricted to the smaller group of nonsmoking snuff users (OR 1.43, 95% CI 1.00–2.05, *p* = 0.048, n = 82) (Table 4). Similar but stronger associations were observed in the group of cases and controls who had never smoked cigarettes, however, not when adjusting for previous exposure to passive smoking (data not shown). In the sensitivity analysis restricted to cases matched to controls included ±2 years of onset (thereby restricting the analysis to cases with onset from 1994 excluding 247 cases consisting of 96% EOMG), the effect size was larger especially in the EOMG group (OR 2.86, 95% CI 1.43–5.72, *p* = 0.003, n = 43, eTable 3), compared with the main analysis (Table 4). The habits of smoking and snuff use have changed substantially in recent years in Sweden, partly driven by a nation-wide ban on smoking in public places including restaurants and bar introduced in 2005. As a result, the frequency of daily smokers has since dropped from 14.8% to 5.8% (2022), whereas the snuff use in women has increased (3.5% 2005 to 7.2% 2022) and slightly decreased in men (22.2% in 2005 to 20.2% in 2022).^34^ We hence hypothesized that the change in behavior might influence the outcome and therefore compared participants with onset before and after 2005. As expected, we observed a 2-fold increase of snuff users at onset in the group with onset after the smoking ban (11.6% compared with 5.8%) (eTable 5).

**Table 4:** Association Between Snuff Use and MG Disease Development

	All			EOMG			LOMG		
	OR	95% CI	*p* Value	OR	95% CI	*p* Value	OR	95% CI	*p* Value
Snuff use at index	1.33	0.99–1.81	0.062	1.61	1.02–2.54	0.039	0.99	0.64–1.54	>0.9
AChR+	1.18	0.80–1.74	0.40	1.42	0.79–2.54	0.24	0.75	0.42–1.32	0.32
Nonsmokers	1.43	1.00–2.05	0.048	1.46	0.83–2.58	0.19	1.17	0.71–1.93	0.53
Ever snuff use	1.14	0.87–1.49	0.36	1.25	0.81–1.93	0.32	1.01	0.70–1.45	>0.9
AChR+	1.00	0.70–1.41	>0.9	0.93	0.52–1.63	0.79	0.94	0.58–1.51	0.79
Nonsmokers	1.14	0.84–1.53	0.41	1.20	0.71–2.02	0.49	0.97	0.65–1.42	0.86

Abbreviations: EOMG = early-onset myasthenia gravis (onset 18–49 years); LOMG = late-onset myasthenia gravis (onset ≥50 years); MG = myasthenia gravis; OR = odds ratio.

All analyses are conditional logistic regression considering the matching variables sex, age at onset, and year of birth. Snuff use at onset and ever snuff use were adjusted for smoking and passive smoke exposure at either onset or ever as well as alcohol consumption.

## Discussion

In this large Swedish nationwide case-control study of prevalent patients with MG and matched controls, we addressed the role of alcohol, smoking, and Swedish snuff on disease risk. We observed a dose-dependent inverse association with alcohol consumption in the whole MG group and in subgroup analyses. Although no strong association was found to neither smoking nor snuff use, an increased risk associated with the use of cigarettes and/or Swedish snuff at disease onset was observed in the EOMG subgroup. This study assesses environmental and lifestyle factors in relation to onset of patients with MG and implicates alcohol as a modifier of disease risk.

We observed a dose-dependent association between alcohol consumption and decreased risk of MG, which is consistent with findings in other autoimmune disorders, such as MS and RA, and may be associated with the immune regulatory effects of alcohol.^24,26,35,36^ Although the effect could not be confirmed in mendelian randomization, the direction of the estimates is in the same direction as observational studies.^37^ Both acute and chronic alcohol consumption has been observed to alter innate and adaptive immune responses.^35^ Decreased absolute B-cell counts have been described in heavy drinkers compared with moderate or light drinkers.^38^ This is of particular interest because B-cell depleting therapy is becoming increasingly recognized as an effective treatment for new-onset MG.^39^ Furthermore, alcohol has been shown to induce thymus atrophy in rats,^40^ a key organ in tolerance induction and MG disease development. Alcohol and its metabolites have also been found to suppress T-cell and T-follicular helper cell function.^41,42^

Regarding tobacco consumption, we observed an association with both smoking and snuff use at disease onset in the EOMG group specifically. Early-onset and late-onset MG differ regarding their HLA associations,^43^ and gene-environment interactions could potentially influence the risk of disease differently in each subgroup. Of interest, one of the main risk loci for EOMG, HLA-DRB1*03, is also associated with anti-Jo1+ myositis, and the risk of anti-Jo1 positivity has been shown to be increased in smokers.^44^ Furthermore, cigarette smoke has been found to alter the composition of lymphocyte subsets, including a decrease of regulatory B-cell counts and immunoglobulin G levels in blood.^45^ The decrease in regulatory B cells is of particular interest because they are important in immunomodulation and suppression of immune responses, and decreased levels have been observed in MG.^46^ In addition, in a small study, nicotine has been observed to reduce Tregs.^47^ In this study, we only observed an increased risk in relation to exposure around onset and no association with intensity or duration (data not shown), and possible mechanisms are hence not certain.

Previous studies suggest a higher frequency of smoking in prevalent patients with MG compared with controls.^22,23^ The prevalence of smokers in the Swedish population is steadily decreasing and was in 2022 5.8% reported by Statistics Sweden and 7% based on an EU-wide survey (lowest of all countries in the European Union).^48^ One could hence speculate that the incidence of EOMG would decrease as the prevalence of smoking decreases. The only study investigating nationwide incidence of MG in Sweden is focusing on the period 2006–2016, where no significant change of EOMG incidence was observed.^6^ Unlike smoking, the use of Swedish snuff has increased slightly, and there were 13.8% daily users in 2022 in Sweden compared with 12.4% in 2004.^34^ However, the trend of Swedish snuff use in women is more striking and has increased more than 2-fold from 3.2% in 2004 to 7.2% in 2022.^34^ Furthermore, use of nicotine products and use of alcohol are often correlated with each other, which could have implications for our results. We observed a higher frequency of drinkers in the LOMG cases smoking at onset (83% compared with 60% in EOMG). As for drinkers in cases who used snuff at onset, we observed similar frequencies in LOMG and EOMG (87% and 88%, respectively). By adjusting for each exposure in the multivariate analysis, we can account for some of this effect. In LOMG, we observed no increased risk of disease development associated with neither smoking nor use of Swedish snuff, which we speculate could be due to too high correlation between alcohol and nicotine use. Since effects are inverse in EOMG, they might rather cancel each other out.

Limitations of the study include using previously collected population controls, which led to some loss of granularity in the exposure variables and inability to adjust for some factors such as geographical location at onset. The retrospective nature of the data will increase the risk for recall bias, and we also observe a longer time from index to inclusion for cases, potentially increasing the recall bias among cases. We have previously attempted to assess recall bias in the cases by investigating how disease duration affects correctly reported onset, diagnosis and thymectomy dates and observed intraclass coefficient correlations of >0.9, indicating excellent reliability.^28^ However, we acknowledge that the recall bias of continuous habits are less readily assessed than those of specific life events. Another limitation is the lack of data on alcohol consumption for the LOMG controls obtained from the EIRA study (n = 251, 25% of LOMG controls). However, in sensitivity analyses excluding EIRA controls, similar results were obtained (data not shown). In addition, data granularity on timing of alcohol consumption in relation to disease onset was reduced because information on alcohol consumption was collected during prespecified 10-year periods. To avoid capturing changes in behavior due to preclinical disease onset, we used the decade before onset as the exposure. Consequently, we entirely lacked data on alcohol consumption in patients with MG with onset before 20 years of age. Furthermore, we did not collect data on possible use of e-cigarettes and could therefore not assess it as an exposure. Although this was a large study of more than 1,000 patients with MG covering more than 40% of the estimated prevalent MG population in Sweden, it might be subject to selection bias, possibly recruiting MG patients with more severe disease phenotypes. Furthermore, the number of cases with exposure to smoking at onset and Swedish snuff use at onset are relatively low, increasing the uncertainty of the results. However, the main findings are persistent throughout subgroup and sensitivity analyses, indicating robustness of the results. Several subgroups analyzed were quite small which is important to have in mind when interpreting the data. We adjusted for possible confounders that were available, but there could possibly be a risk for residual confounding, either due to low granularity for the factors adjusted for or due to potential confounding factors not adjusted for. The serologic data are not complete, and we lacked data on n = 316 cases. There is some risk for misclassification bias in the AChR− group because new more sensitive methods might have been able to detect antibodies if these patients would be retested. However, the frequency of AChR+ patients in the subset with available serology was 85%, similar to what has been previously observed. Owing to the lack of complete AChR status, we only classified EOMG and LOMG based on age at onset and did not take AChR status into account.

In conclusion, we find alcohol consumption to have an inverse association with disease development, that is in line with previous research in other autoimmune diseases such as RA, MS, and SLE and adds support to a negative correlation with autoimmunity in general.^24-26,41,42^ From a public health perspective, the negative effect on other health outcomes such as the risk of substance abuse and cancer development may however outweigh the benefits, especially among heavy drinkers.^49^ Smoking and use of Swedish snuff were only identified as risk factors at onset, primarily for patients with EOMG and especially when restricting the analysis to AChR+ patients, possibly implicating pathogenic mechanisms in nicotinergic signaling. In both MS and RA gene-environment, interactions between tobacco use and HLA-genes have been shown to significantly increase disease risk.^32,50^ Future studies dissecting the effect of alcohol, tobacco use, and the interaction with genetic variants on MG disease development and on severity would be of great interest.

## Glossary

AChr+: acetylcholine receptor positive
EIMS: Epidemiological Investigation of Multiple Sclerosis
EIRA: Epidemiological Investigation of Rheumatoid Arthritis
EOMG: early-onset MG
GEMG: Genes and Environment in Myasthenia Gravis
GEMS: Gene and Environment in Multiple Sclerosis
IMSE: Immunomodulation and Multiple Sclerosis Epidemiology
LOMG: late-onset MG
MG: myasthenia gravis
MS: multiple sclerosis
OR: odds ratio
RA: rheumatoid arthritis
SLE: systemic lupus erythematosus
TAMG: thymoma-associated MG
